# An Unusual Case of Foreign Body Lodged in the Laryngopharynx of Neonate with Esophageal Atresia

**Published:** 2016-01-01

**Authors:** Rahul Gupta, Manisha Saxena, Rozy Paul, Sharan Gubbi, Praveen Mathur

**Affiliations:** 1 Department of Paediatric Surgery, Sawai Man Singh (SMS) Medical College Jaipur, Rajasthan, India; 2 Department of Anaesthesia, Sawai Man Singh (SMS) Medical College, Jaipur, Rajasthan, India; 3 Department of Anaesthesia, Bhagwan Mahavir Cancer Hospital & Research Center (BMCHRC), Jaipur, Rajasthan, India

**Keywords:** Esophageal atresia, Foreign body, Laryngopharynx, Neonate, Red rubber catheter

## Abstract

A blunt‑tipped red rubber catheter is used to confirm the presence of esophageal atresia in any newborn with drooling of saliva and frothing from the mouth. Failure to pass it beyond 10cms into the esophagus is considered diagnostic. We here in report an extremely rare case of broken tip of red rubber catheter lodged in the laryngopharynx of 2-day-old neonate of esophageal atresia with distal tracheoesophageal fistula. During endotracheal intubation foreign body was accidentally removed.

## CASE REPORT

A 2-day-old female neonate presented with drooling of saliva and frothing from the mouth. She was referred from another hospital where a diagnosis of esophageal atresia was made before she reached our institute. On examination, the patient was stable, respiratory rate-60/min, and pulse rate-150/min. She had low birth weight (1.8 kg). Chest examination revealed conducted sounds. There was mild abdominal distension. After oropharyngeal suctioning, a no.10 red rubber catheter confirmed the presence of esophageal atresia. The patient was taken to radiology suite for performing a radiograph with red rubber catheter in place. The radiograph revealed red rubber catheter in upper pouch and a cylindrical foreign body in the upper aero-digestive tract, which was extremely unusual (Fig.1). It also showed dilated stomach and gaseous abdomen, confirming the diagnosis of EA with TEF. The patient was taken to the operation theatre for removal of foreign body. For securing the airway the patient was placed in supine position to enable laryngoscopy and endotracheal intubation was attempted. During the procedure endotracheal tube was stuck inside the lumen of broken tip of red rubber catheter and it was removed accidentally (Fig.2). Laryngoscopy was again performed to rule out any injury. A fresh red rubber catheter was reinserted to reconfirm esophageal atresia. As the patient was stable and there was no evidence of bleeding, a decision was taken to proceed with primary repair of EA with TEF. Right postero-lateral extrapleural thoracotomy was performed. The procedure was accomplished without any complication.

**Figure:**
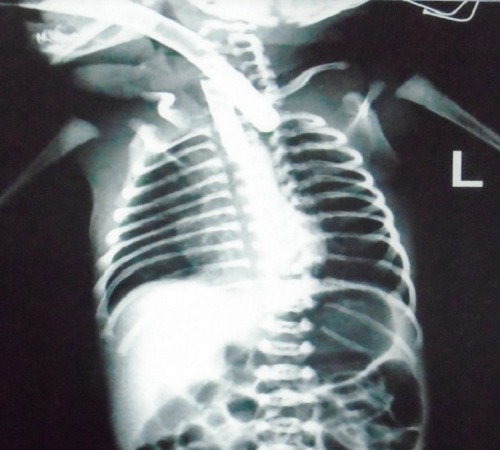
Figure 1: The radiograph revealed red rubber catheter in upper pouch and a cylindrical foreign body in the upper aero-digestive tract.

**Figure:**
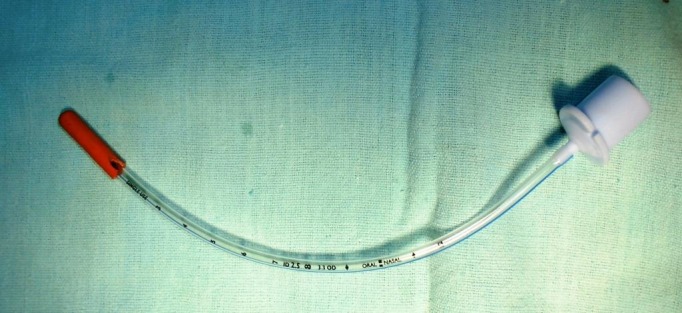
Figure 2: Broken tip of red rubber catheter engaged at the tip of endotracheal tube which was removed accidentally.

## DISCUSSION

Foreign bodies in neonates are extremely rare [1-3]. In the earlier case reported in the literature, the tip of red rubber catheter broke intraoperatively and it was retrieved with the help of a Magill’s forceps under endoscopic vision, lodged high in the nasopharynx [1]. In the present case, the neonate was received with the foreign body lodged in the laryngopharynx, revealed by the radiograph and finally broken catheter tip was discovered and removed accidentally during endotracheal intubation. It is observed that eye of red rubber catheter is potential site for tear and breakage and it was consistent finding in both the cases. The predisposing factor could be due to inferior quality of the catheter or harsh climatic conditions in our country. We propose that red rubber catheter should be carefully checked (for any tear/fracture) every time it is inserted into the upper pouch.

## Footnotes

**Source of Support:** Nil

**Conflict of Interest:** Nil
